# βCaMKII regulates bidirectional long-term plasticity in cerebellar Purkinje cells by a CaMKII/PP2B switch mechanism

**DOI:** 10.1186/1471-2202-15-S1-P58

**Published:** 2014-07-21

**Authors:** Thiago M Pinto, Maria J Schilstra, Volker Steuber, Antonio C Roque

**Affiliations:** 1 Departamento de Física, FFCLRP, Universidade de São Paulo, Ribeirão Preto, SP, 14040-901, Brazil; 2 Science and Technology Research Institute, University of Hertfordshire, Hatfield, Herts, AL10 9AB, UK

## 

It is thought that modifications in the strength of synaptic connections onto cerebellar Purkinje cells (PCs) such as long-term depression (LTD) and long-term potentiation (LTP) contribute to learning. Repeated paired stimulations of parallel fibres (PFs) and climbing fibre (CF) evoke high-amplitude calcium signals that lead to LTD in PCs, while LTP is induced by low-amplitude calcium pulses as a result of PF input alone. Although the events following these stimulation protocols are becoming clearer, the precise interactions of the molecular machinery underlying LTD and LTP induction remain something of an enigma. Calcium-calmodulin dependent protein kinase II (CaMKII) is an important component of the signalling network that is responsible for cerebellar plasticity in PCs. The CaMKII holoenzyme contains isoforms such as αCaMKII and βCaMKII. Experiments with Camk2b knockout mice that lack the β isoform of CaMKII have demonstrated that βCaMKII regulates the direction of plasticity at PF-PC synapses [1]. Stimulation protocols that induce LTD in wild-type mice, which contain both α and βCaMKII, lead to LTP in knockout mice without βCaMKII, and vice versa.

In previous work, we have developed a kinetic simulation of the phosphorylation and dephosphorylation of AMPA receptors by CaMKII and protein phosphatase 2B (PP2B) to investigate how βCaMKII controls bidirectional plasticity in cerebellar PCs [2]. This bidirectional plasticity model was based on our earlier model of CaMKII activation [3,4]. Because the βCaMKII, but not αCaMKII, isoforms can bind to filamentous actin (F-actin), we have included the binding of F-actin to CaMKII to simulate plasticity induction in wild-type mice, whereas the F-actin binding was omitted in the knockout mice that lack the β isoform. Our previous simulation results have demonstrated that the binding of F-actin to βCaMKII can contribute to the regulation of bidirectional plasticity at PF-PC synapses, as suggested by Van Woerden and collaborators [1]. However, we have investigated the induction of bidirectional plasticity only in the presence of calcium signals, i.e. during the early phase of LTD and LTP. But the experimental observations in [1] have demonstrated that the bidirectional plasticity is also observed after the offset of paired PF and CF inputs and PF input alone. To analyse the persistent AMPA receptor phosphorylation after calcium stimulation protocols that last 5 min, we modified our previous model [2] because it did not simulate the CaMKII dephosphorylation, and PP2B was rapidly inactivated with the interruption of calcium signals. Here, we allow the CaMKII subunits in the autonomous form, which are not bound to calcium-calmodulin but still retain some activity levels, to be dephosphorylated and switch to the inactive state of CaMKII. The kinase is therefore gradually inactivated after calcium stimulation stops. Moreover, calcium-calmodulin slowly dissociates from PP2B, leading to a gradual deactivation of PP2B.

Results of computer simulations of our mathematical model suggest that the balance of CaMKII-mediated phosphorylation and PP2B-mediated dephosphorylation of AMPA receptors determines whether LTD or LTP occurs at the PF-PC synapse. PF activity alone evokes PP2B concentration levels higher than CaMKII, leading to LTP induction. Instead, LTD occurs when the CaMKII concentration levels surpass the PP2B concentration in response to the paired activation of PF and CF. Moreover, our recent simulation results replicate the experimental observations by Van Woerden et al. [1] not only in the early phase of LTD and LTP as shown in [2], but also in their intermediate and late phases after the termination of PF and CF stimulation and PF input alone. The mechanisms observed in our previous simulations with 5 min duration are also observed in simulations that last 100 min. The loss of F-actin binding in the knockout mice enhances the availability of active CaMKII when compared to the wild-type mice at low calcium concentrations. This favours the induction of LTD rather than LTP. In contrast, the reduction in the CaMKII concentration in the knockout mice leads to AMPA receptor dephosphorylation by PP2B for high calcium concentrations, leading to the induction of LTP instead of LTD. We again demonstrate that the binding of F-actin to βCaMKII can indeed contribute to the control of bidirectional plasticity at PF-PC synapses, as suggested in [1,2].
